# Designing community facing tools for green stormwater infrastructure and groundwater monitoring: a model of interdisciplinary action research

**DOI:** 10.1088/2515-7620/ae0877

**Published:** 2025-10-07

**Authors:** Brendan F O’Leary, Colleen Linn, Darrin Hunt, Brittanie Dabney, James Hartrick, Kate Ekhator, Natalie Lyon, Carol J Miller, Rahul Mitra, Michelle Serreyn, Sadaf Teimoori

**Affiliations:** 1 Department of Civil and Environmental Engineering, Wayne State University, Detroit, MI, United States of America; 2 Department of Biological Sciences, Wayne State University, Detroit, MI, United States of America; 3 Department of Anthropology, Wayne State University, Detroit, MI, United States of America; 4 Department of Environment and Sustainability, Shenandoah University, Winchester, VA, United States of America; 5 Limnotech, Ann Arbor, MI, United States of America; 6 RESPEC, Roseville, MN, United States of America; 7 Department of Communication, Wayne State University, Detroit, MI, United States of America; 8 Michigan Department of Natural Resources, Detroit, MI, United States of America; 9 Jacobs Solutions, Inc, Royal Oak, MI, United States of America

**Keywords:** GSI, hydrogeology, groundwater contaminants, urban hydrology, water quality, citizen science, microplastics

## Abstract

Anthropogenic stress associated with urban development can change groundwater hydrology and introduce contaminants that may compromise its utility. Green Stormwater infrastructure (GSI) alters local landscapes and uses natural flora to manage stormwater and mitigate flooding, but it may also influence groundwater hydrology, and contaminant fate and transport. This multidisciplinary project engaged with community groups to develop and implement a low-cost, citizen-led groundwater monitoring program at the GSI sociotechnical interface in Detroit, MI (USA). GSI installations were constructed at a historic property in urban Detroit, MI. Groundwater monitoring wells were installed throughout the property and at a nearby community-maintained, long-term reference GSI for comparison. From April to October 2021, groundwater and contaminant levels in wells and adjacent soil were assessed using affordable products that citizen science groups could easily employ. Implementing this monitoring program, we identified the significant role GSI plays in increasing groundwater recharge, nutrient and coliform levels. Data collected show slight increases in hydraulic head, especially near GSI, after installation, enhancing groundwater recharge. Before and after MODLOW models of the site show that the GSI also impacted local groundwater flow. Soil nitrite was higher, and *E. coli* was lower near GSI installations. However, total coliform counts increased over time near GSI. These results have implications for hyperlocal flooding concerns and pollution monitoring in a post-industrial urban setting. Partnering with community groups allowed researchers to engage with local concerns and provided opportunities to share outputs for community-facing planning and education. This article discusses the results of groundwater monitoring and testing and explores how multidisciplinary research can facilitate integrating academic research with local concerns. We conclude by discussing how future research can deepen these connections for ongoing knowledge advancement and exchange regarding urban water challenges.

## 1. Introduction

Water is a critical resource in urban areas, but it can be difficult to characterize given the high degree of hydraulic modifications in urban settings (Bach *et al* 2014). Anthropogenic disturbance, manipulation of urban watersheds, and the development of municipal water and wastewater treatment plants have created human-hydrologic systems that flow between natural, built, and urban environments (Claessens *et al* 2006, Gessner *et al* 2014, Shaad and Burlando 2019, Yu *et al* 2019). It is particularly important for researchers to pay attention to groundwater flows and contamination because it plays a critical and often underappreciated role in global water systems, ecosystems, and human well-being, supplying about 50% of the world’s population (United Nations 2022). Despite its interconnection with surface water and ecosystems, groundwater is often ignored and harder to monitor, leading to over-extraction, contamination and depletion, which disproportionately impacts low-income and other marginalized communities (Jia *et al* 2020, Li *et al* 2021, Sophocleous 2002). Therefore, it is critical to recognize the role humans play in the groundwater cycle, including defining hyper-local hydrosocial contexts to address the confluence of water-related concerns found across urban and semi-urban environments (Linton and Budds 2014). Our study provides a useful model for interdisciplinary action research that designs community-facing tools and scientific interventions for groundwater monitoring and installation of green stormwater infrastructure to help mitigate flooding and environmental pollution.

Action research, grounded in the work of pragmatic theorists Kurt Lewin, John Dewey and others, can broadly be defined as a systematic approach to solving ‘real-world’ problems by integrating research and action (Lewin 1946, Marrow 1969). Far from being atheoretical, action research is guided by empirical research and data collection in specific contexts, which is often conducted by partnerships between researchers and impacted communities and other stakeholders (Adleman 1993). Inherently iterative, action research cycles through phases of planning, action, analysis and reflection, resulting in solutions that were both feasible and empowering for impacted communities (Cohen *et al* 2017). While sometimes critiqued for its lack of generalizable findings, action research is deeply grounded in data, local contexts, and lived experiences, and can both improve existing systems while unearthing innovative ideas for comparable situations elsewhere (Altrichter *et al* 2002, Checkland and Holwell 2007). Adopting an action research framework for monitoring groundwater for environmental pollution is crucial, we argue, because it allows for place-based understanding and deeper engagement with local factors that shape hydrosocial systems (e.g., land use, infrastructure, pollution sources, community water management practices). Designing scientific research grounded in and building from impacted communities’ long-term experiential knowledge (e.g., flooding patterns, contamination events) can identify especially relevant variables, validate findings, and interpret anomalies, resulting in feasible solutions and practical interventions that are more likely to be embraced by stakeholders (Jadeja *et al* 2018, Shalsi *et al* 2022). In contrast, top-down water management solutions and research outcomes that are entirely derived deductively often face a crisis of trust and legitimacy from local stakeholders, or it is unclear to communities of interest how exactly they should adapt suggested (supposedly generalizable) solutions to their local systems (Foster and van der Gun 2016). Moreover, action research embraces an ethos of radical interdisciplinarity, pushing researchers to encourage systems thinking and bridge natural-versus-social science silos, and generate holistic and innovative solutions that might have been beyond traditional disciplinary boundaries. These concerns, voiced by a growing chorus of groundwater researchers from various disciplines (e.g., Barthel and Seidl 2017), are especially relevant in urban areas populated by low-income and underserved communities of color (such as Detroit, MI), who are disproportionately impacted by groundwater contamination and flooding, and clamor for research partnerships that deliver feasible, innovative and low-cost solutions. Frameworks such as the Green Infrastructure Rapid Assessment (GIRA) protocol (Meixner *et al* 2021) and community science approaches for environmental monitoring (Eitzel *et al* 2017) demonstrate how peer-reviewed research can provide tools for impacted communities to investigate and learn about environmental conditions directly affecting them.

In the following sections, we overview some key research on groundwater management problems in Detroit (the site of our action research) and how green stormwater infrastructure can address them. Next, we describe our interdisciplinary methods of data collection and analysis, and detail our findings. Finally, we discuss some key contributions of our study and, by way of conclusion, reiterate its importance.

## 2. Groundwater and green stormwater infrastructure: a hyperlocal approach

Cities in the U.S. Midwest, like Detroit, grapple with managing outdated infrastructure, increased heavy rain frequency, neighborhood flooding, and limited groundwater knowledge (Mozola 1969, Rogers 1996, Sampson *et al* 2019). Detroit’s high-water table also contributes to pollution from surface water runoff and household flooding. These issues collectively hinder the city’s capacity to effectively manage rainwater and reduce neighborhood flooding and stormwater runoff into the Rouge and Detroit Rivers. In response, green stormwater infrastructure (GSI) has emerged as a cost-effective solution to alleviate the rainfall burden on neighborhoods and the city’s combined sewer system. To date, over 200 GSI projects have been implemented across Detroit (Detroit Stormwater Hub 2024). While GSI installations have alleviated a portion of the water burden on grey infrastructure (Steis Thorsby *et al* 2020), flooding still impacts residents, leading to psychosocial distress, socioeconomic hardship, and human health impacts (Sampson *et al* 2019, Larson *et al* 2021).

The scarcity of data regarding groundwater quality, quantity, flow, and transport presents a critical gap in urban planning and design, complicating the effective implementation of green stormwater systems in Detroit. Detroit’s groundwater quality is significantly influenced by the city’s industrial legacy and urban development, particularly prior to modern environmental regulations. Furthermore, the groundwater knowledge deficit raises health concerns related to the poorly understood transport of groundwater pollutants throughout the Great Lakes Basin (Warner *et al* 2015). Emerging contaminants, such as microplastics, volatile organic compounds (VOCs), and per- and poly-fluoroalkyl substances (PFAS), can infiltrate groundwater and accumulate in soils, potentially contributing to adverse human health effects (Miller *et al* 2020, Huang *et al* 2021, Llewellyn *et al* 2024).

The prevalence of GSI in urban environments like Detroit presents a valuable opportunity to investigate how these systems influence groundwater quality—addressing the current scarcity of groundwater-quality data—while yielding insights into contaminant transport, infiltration dynamics, and the potential for GSI to either mitigate or mobilize pollutants within the subsurface. The conveyance of water away from GSI and contaminant movement through GSI and groundwater is not well-studied. Understanding GSI impacts on contaminant and groundwater flow is important for urban water budgets, and its potential impact on water quality and environmental safety (Townsend *et al* 2003, Dubrovsky and Hamilton 2010). While GSI can reduce the concentration of contaminants, its effectiveness is uncertain, as nitrogen is highly mobile, and phosphorus is transported in suspended sediments (Kornelsen and Coulibaly 2014). Common indicators of groundwater contamination include nutrients (nitrate, nitrite, phosphate) and fecal coliform levels (Pedley and Howard 1997, Atherholt *et al* 2003, Wick *et al* 2012). In the Great Lakes region, groundwater contamination contributes to fecal coliform transport to surface waters and hinders brownfield redevelopment (Kaufman *et al* 2003, Howard and Gerber 2018). Assessing GSI’s impact on groundwater can extend protections to surface water bodies critical for regional water security. The Detroit River, for example, is an international waterway that supplies drinking water to approximately 6.2 million people across southeastern Michigan and southwestern Ontario (Michigan Department of Environmental Quality 2008), illustrating how localized groundwater–surface water interactions can have far-reaching and transboundary public health and environmental implications.

Beyond its role in protecting critical water resources like the Detroit River, GSI also delivers important co-benefits at the neighborhood scale. While the primary goal of GSI is to reduce the impacts of heavy rainfall, its presence in urban communities can also enhance environmental quality and improve neighborhood livability. Incorporating GSI into neighborhoods can increase residents’ involvement in their immediate surroundings, including awareness of how the built environment impacts urban hydrology issues, like neighborhood flooding (Barclay and Klotz 2019). Community gardens, green streets, and urban wetlands are often used as educational tools, connecting people with nature and encouraging them to take part in local conservation efforts. Neighborhood green spaces can promote community engagement, and environment-related public health education (Kondo *et al* 2018, Carmichael *et al* 2019) while creating aesthetically pleasing, healthy environments. However, successful long-term maintenance of GSI often requires an active local volunteer base to ensure installations function properly year-round. Approachable and cost-effective tools can help community groups to effectively engage with and manage urban hydrology concerns.

While many previous studies on green stormwater infrastructure (GSI) have emphasized the importance of community input and engagement mechanisms in planning and implementation (Adib and Wu 2020, Abera *et al* 2021, Heidari *et al* 2023), few provide communities with direct tools to assess the hydrological and water quality impacts of GSI installations (Horner *et al* 2002, Spatari *et al* 2011). Our study advances the field by offering a low-cost, accessible resource framework that empowers community groups to evaluate how GSI alters local groundwater flow and quality. By integrating simplified monitoring strategies with educational materials, this framework fills a critical gap between community engagement and environmental data collection, promoting deeper understanding and stewardship of local water systems.

This paper presents findings from a multidisciplinary research project focused on the intersection of Detroit’s groundwater (GW) and GSI. We established a network of community-based GW monitoring stations near GSI sites in two urban neighborhoods. Our approach offers a cost-effective method for installing GW monitoring stations that citizen science groups can utilize to enhance understanding of GW flow and quality. In collaboration with local organizations, we also designed educational resources to increase community awareness of factors affecting urban watersheds. Our goal, therefore, was to include community partners’ perspectives and concerns at every phase, leading to long-term systemic resilience and sustainability of the urban neighborhoods (Mitra 2020), rather than creating one-off or temporary solutions. The GW stations enabled monitoring of urban contaminants and allowed for low-cost, rapid water quality assessments. Partnering with community groups also provided valuable opportunities to address local concerns and share data for neighborhood planning. Our main objectives were to: 1) expand knowledge of Detroit’s GW, 2) assess GSI’s impact, 3) foster community partnerships on water issues, and 4) develop a low-cost GW monitoring solution.

## 3. Methods

**Figure 1.**
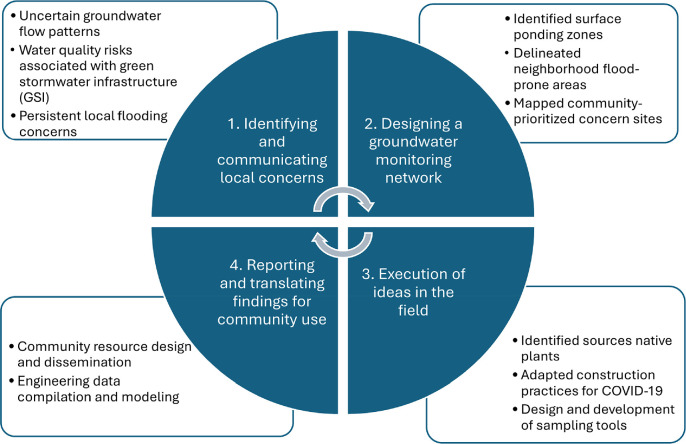
Research roadmap illustrating the study design process, including community resource development and engineering data and modeling. The central circular element represents the main interactive and iterative framework, while surrounding boxes highlight key variables and components used in our study.

Our methodological approach was structured around a community-informed, iterative research design comprising three interconnected phases: (a) identifying and communicating local concerns related to GW and GSI; (b) designing a groundwater monitoring network tailored to these concerns; and (c) implementing the network at selected field sites. Although these phases were not always sequential, they informed one another throughout the study. Community engagement was embedded throughout both the design and implementation phases, including regular discussions with local partners to ensure the monitoring strategy reflected neighborhood-specific priorities. Feedback from field implementation directly informed the development of outreach materials and data communication products, completing a full cycle of co-produced knowledge and methodological refinement. Figure 1 presents a research roadmap illustrating the study design process, which integrates community resource development with engineering data and modeling.

### 3.1. Understanding and communicating local concerns

The concurrent issues of outdated water infrastructure, increased rainfall, and low GW knowledge necessitate a range of solutions that benefit Detroit households and water bodies (Schilling and Logan 2008, Berkooz 2011, Nassauer and Raskin 2014). GSI when managed properly, can mitigate neighborhood flooding, increase GW infiltration, and reduce the burden on combined sewer systems during rain events (Sohn *et al* 2020). GSI installations can also act as sites of knowledge sharing and community integration, where professional and volunteer expertise are needed to install and maintain GSI.

Our multidisciplinary research team, drawing from Civil Engineering, Biological Sciences, Urban Planning, Communication, and Anthropology, sought multiple partnerships to accomplish the technical and community-facing aspects of the project. University partnerships included department courses, student university groups, and university departments, to coordinate building a research bioswale, setting up a GW monitoring station, and installing an informational sign on university grounds. Two university classes from multiple departments (Biology, Communication, and Civil and Environmental Engineering) engaged with the team to construct communication tools and quantitative assessments. Student university groups, including the DBN, connected team members with local plants for the campus bioswale. The team hosted community members for GSI tours during several events, including a regional conference, the 2021 GLBD; attended local meetings, such as WS community outreach event, and presented project outcomes at the international 2022 Joint Aquatic Sciences Meeting in Grand Rapids, MI. These relationships guided the project’s public-facing outputs, including disseminating educational content, and facilitated regular access to off-campus GW monitoring field sites.

The university classes contributed to background research and produced deliverables that were used to share knowledge with our community partners. They included a senior capstone course in the Communication department and a 1-credit elective seminar course through a research trainee program funded by the National Science Foundation. Several research team members were a part of the trainee program and led the seminar. The seminar produced a land use map for Chandler Park, which identified flood-prone areas and ways to reduce run-off on their grounds. The capstone course produced communication media products for our community partners, Chandler Park Conservancy (CPC) and East Side Community Network (ECN), on four key topics related to the project (groundwater, groundwater quality, combined sewer systems, and water budgets). Communication materials, including infographics and whiteboard animations, were co-developed with community partners to ensure clarity and accessibility. Decisions regarding format and content were made collaboratively through virtual meetings. Finalized products were provided to our community partners to use for planning purposes and outreach and education initiatives at their discretion. We also distributed the products at various public events, listed above.

### 3.2. Design of a groundwater monitoring network for sampling and analysis

**Figure 2.**
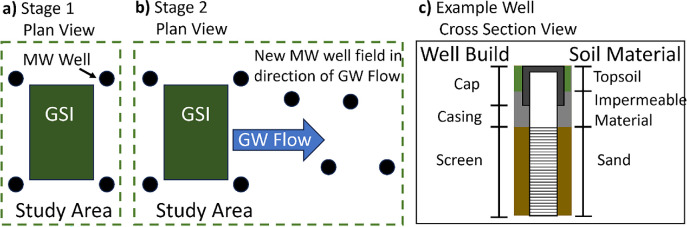
Green stormwater infrastructure (GSI) and groundwater well design, displaying (A) Phase 1 plan view of well placement around GSI, (B) Phase 2 plan view with extended well placement, and (C) example cross-sectional well design.

To assess GW around GSI, the technical challenge was to develop a scalable, low-cost, community-based GW monitoring network to measure flow and quality at a neighborhood scale. Our solution involved small, easily deployable monitoring wells in urban environments, resulting in a modular, adaptable, and cost-effective system tailored to the varied conditions of GSI and urban soils. The monitoring system was designed in two stages: Phase 1 involved installing GW wells adjacent to the proposed GSI to establish baseline data on hydraulic head and GW chemistry. Phase 2 extended the well field to assess changes in both flow patterns and GW chemistry over time (figure 2). In Detroit, where GW levels are shallow due to geological conditions (Rogers 1996), hand augering significantly reduced installation costs. Wells were installed at a depth of approximately 10 feet using 2-inch PVC well screens and casings. Sand was used to backfill the annular space around the screen, while the top two feet were sealed with bentonite to prevent surface water intrusion (figure 2). Flush mount caps ensured the wells were unobtrusive and minimized neighborhood disturbance during ongoing monitoring activities (Striggow 2018).

GW flow direction was determined using the free EPA 3PE tool to calculate flow vectors at both field sites (Beljin *et al* 2014). This tool, applied to Phase 1 wells, employed a three-point solution method to estimate horizontal hydraulic gradients and GW velocities, enabling quick visualization of hydraulic gradient and velocity vectors. Using the urban water budget model from Teimoori *et al* (2021), a simple urban water budget was developed for each GSI location, based on data from the GW monitoring wells and open-source USGS weather data (equation (1)). Key parameters in the mass balance include the change in groundwater storage (ΔS), precipitation (P), evapotranspiration (ET), runoff (R) from the site, drainage (D) through subsurface flows, and where available, changes in soil moisture (*θ*), all measured in cubic meters (m^3^)

\begin{eqnarray*}\pm {\mathrm{\Delta }}{\mathrm{S}}={\mathrm{P}}-\left({\mathrm{ET}}+{\mathrm{R}}+{\mathrm{D}}\right)\end{eqnarray*}

Additionally, to validate our design, we developed a GW model to evaluate changes in GW flow. Data from the wells enabled us to assess how GSI influenced local GW dynamics.

To assess the impact of GSI on contaminant movement, wells were monitored for microbes, nutrients, and microplastics. GW and soil samples were taken together. The proposed methods were a disposable polyethylene bailer and a peristaltic pump (Vail 2013). Our team sampled GW by lowering a plastic tube to the bottom of the well and extracting water into a glass jar with a peristaltic pump. Soil samples were composites of three auger pulls (≤10-inch depth) randomly collected near GW monitoring wells. Bulk soil and benthic sediment were placed in 400 ml glass jars and stored at 4 °C until processing.

Laboratory testing for nutrient analytes and microbes can be costly and limit the scope of community-led biomonitoring programs. To explore low-cost, accessible soil and GW testing options, nutrients, coliforms, and physicochemical parameters were evaluated using products manufactured by Hach, LusterLeaf, 3M, and Difco. These products can be easily employed by community-led citizen science soil and GW monitoring programs. LusterLeaf and Hatch kits were used to test GW and soil samples for nutrient analyte levels (nitrite, nitrate, and phosphorus) (Ormaza-González and Villalba-Flor 1994). *E. coli* and total coliforms were quantified using 3M Petrifilm, Hatch m-ColiBlue24 broth, and Difco™ mEI Agar was used to detect Enterococci (Hach Company 1982, Vail *et al* 2003). A 1:10 dilution was created using water and soil extracts, and 1 ml of the dilution was placed on *E. coli* and Enterococci Petrifilms. Similar dilutions were used when plating Hach and Difco broth and agar. Microplastics were separated from GW and soil using high-density salts (NaI, and NaCl) and inspected with fluorescent microscopy.

At the time of sampling, water quality values, including pH, conductivity (μs/cm), temperature (°C), dissolved oxygen (mg/l), and turbidity (NTU), were documented using a multiparameter probe. Soil pH, moisture, fertilizer, and sunlight penetration were detected using a LusterLeaf Rapitest Tester Electronic 4-Way Analyzer. The YSI parameter probe was calibrated monthly using laboratory standards, while Hach and Difco tests for nutrient analyte and microbe concentrations were calibrated to zero by the manufacturer before shipping.

### 3.3. Implementation in field sites in detroit

**Figure 3.**
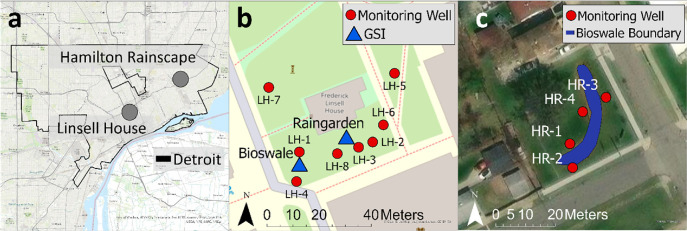
Aerial view of (A) two field sites; (B) Linsell House with Green stormwater infrastructure (GSI) and wells; and (C) Hamilton Rainscape with GSI and wells.

The project team identified two locations for fieldwork (figure 3(A)). At the first location, Linsell House (LH), the team built a research bioswale, rain garden, and GW monitoring wells adjacent to a former residential building on the southern edge of the university’s Detroit campus. The second location, Hamilton Rainscape (HR), was a preexisting bioswale on the city’s east side operated by ECN. The HR location emerged from community plans to address blight and stormwater issues. Our project was initially scoped for implementation at LH and we successfully completed our Phase 1 and Phase 2 designs at this location. To validate our approach, we applied a GW simulation model at LH, providing a comprehensive understanding of GW flow around the GSI installation. While the COVID-19 pandemic limited on-campus engagement, we were able to connect virtually with community groups and include the HR dataset. The location and design elements were selected through a community engagement process that brought together residents and designers throughout the project’s development. All data is presented; however, Phase 2 and modeling were confined to LH. GW wells were installed and monitored from April 19 to October 31, 2021, at LH and May 14—October 21, 2021, at HR.

At the LH site (figure 3(B)), four wells were installed in early summer (LH 1–4) before constructing a bioswale and rain garden. These wells were located directly adjacent to where the GSI would be installed but were installed pre-construction to establish baseline data. Following construction, an additional four wells (LH 5–8) were added, away from the GSI, allowing us to contrast its effects on GW contaminant concentrations. At the HR site (figure 3(C)), which had been established for several years, four wells (HR 1–4) were installed to monitor the area’s GW conditions. All wells were outfitted with Solinst Levelogger^®^ 5 Water Level Dataloggers for continuous monitoring of hydraulic head and GW temperature.

The modeling work relied on GMS-MODFLOW 2000 (Aquaveo 2023), a robust and widely used GW modeling tool. MODFLOW operates using the finite-difference method to simulate the movement of GW through porous media, making it particularly suited for studies like this, where water flows through different soil layers is key (McDonald and Harbaugh 1988). In the LH model, the system was simulated under steady-state conditions, meaning that the model assumed GW flow conditions remained constant over time. This approach allowed for a clearer understanding of how groundwater would respond to the introduction of GSI features over an extended period.

In the summer and fall of 2021, we sampled soil and GW from 12 monitoring wells at the two GSI locations, LH and HR. GW and soil samples were collected from eight wells at LH: four that were installed prior to GSI construction (LH 1–4) and four afterward (LH 5–8). Pre-construction wells were sampled once in April 2021, and all wells (LH 1–8) were sampled in July, August, and September 2021. Four wells installed at HR (HR 1–4) were sampled once a month in May, August, September and October 2021.

To understand changes in GW and soil caused by GSI construction, nutrient, coliform, microplastic, and physicochemical data from LH1–4 were compared to LH 5–8, and HR 1–4 using a series of analyses of variance (ANOVA) (n = 24). If the assumptions of the ANOVAs were not met, non-parametric Kruskal–Wallis (KW) rank sum tests would be used. Pairwise comparisons were conducted on ANOVAs and KWs using Tukey’s HSD and Dunn’s tests. To identify how GSI installation impacts GW and soil over time, linear regression analyses were conducted on pooled data across LH 1–4 (n = 4). When significant differences in LH, MW 1–4 data over time were detected, LH 5–8 wells were also analyzed to determine if GSI may be driving these changes.

## 4. Results

### 4.1. Community-university knowledge sharing and learning

**Figure 4.**
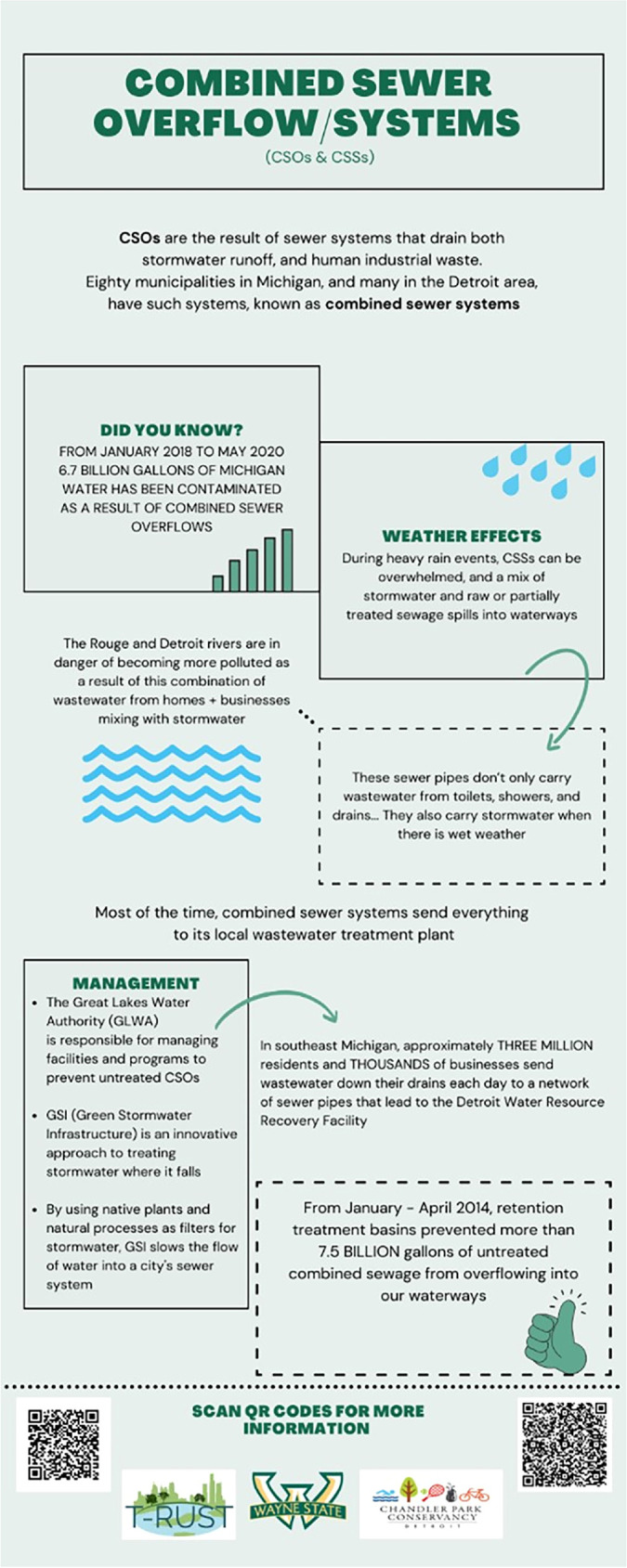
Example of communication infographics developed to discuss urban water challenges.

The community engagement components of this research resulted in several educational and science communication deliverables, a successful partnership between university and community groups, and research team members participating in community events to share information about urban water concerns. The deliverables included four infographics (figure 4) and corresponding videos on the topics of GW, GW contamination, combined sewer systems, and urban water budgets. Topics were selected by members of the research team, and products (figure 4) were developed by senior undergraduate students in a communications capstone course. Research team members guided students on informational content and selecting evidence-based theoretical frameworks while students took the lead in designing the items. Three widely used theories were used—the health belief model, elaboration likelihood model, and the narrative paradigm—to ensure that resulting products would be effective. In line with the health belief model (for a review, see Janz and Becker 1984), we emphasized the six elements of audiences’ perceived susceptibility to problems stemming from inadequate GW management (e.g., flooding), perceived severity of these problems (e.g., property damage), perceived benefits of installing GSI (e.g., better long-term health), perceived barriers against installation (e.g., aesthetic factors), cues to action (e.g., simple maintenance of GSI), and self-efficacy (e.g., community members can help prevent long-term flooding). The elaboration likelihood model (Petty and Cacioppo 1986) enabled us to design the products keeping in mind how different individuals perceive and process information, depending on their preexisting knowledge on a topic, motivation to learn more, ability to pay attention, temporal and spatial proximity, and salient identities. Finally, guided by Fisher’s (1985) narrative paradigm, we wrote scripts for the short informational videos that emphasized narrative fidelity and coherence, paying attention to the logical sequence of events in the script/video, introduction, and development of scenes and characters, reasoning patterns, credibility of the narrators, and overarching structure of the narratives both within each video and connecting the videos together in a single series. Drawing on these frameworks, the developed infographics and whiteboard animation videos connected the ways in which local infrastructure, urban flooding, and GSI presence impact urban areas and Detroit neighborhoods and urged residents to learn more about both the problems and possible solutions. These products were then given to our community group partners and were utilized by the research team at public events and discussions.

**Figure 5.**
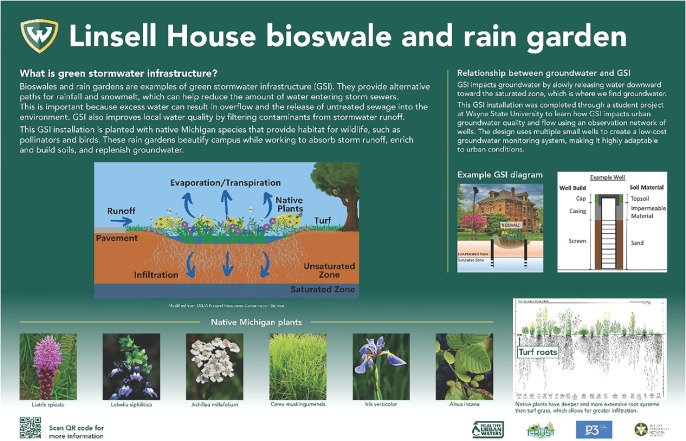
Linsell House green stormwater infrastructure educational sign.

Additionally, a 1-credit elective course worked to produce a land use map that identified areas of Chandler Park that identified flood-prone areas and ways to reduce stormwater run-off. This map was presented to representatives of Chandler Park for their consideration as they planned future park amenities. Finally, the research team designed and installed an informational sign (figure 5) about the benefits of GSI on the university’s campus adjacent to the LH, where the researchers’ on-campus GW monitoring station and bioswale are located.

The city of Detroit’s continued investment in GSI over the past decade suggests its local familiarity will continue to grow. The prevalence of neighborhood GSI provides an opportunity to engage the public and broaden community-level knowledge about concurrent urban water concerns, such as heavy rainfall, GW quality, grey infrastructure, and flooding (Barclay and Klotz 2019). In line with the ‘integrated approach’ to incorporating GSI in underserved communities favored by Garcia-Cuerva *et al* (2018), our design/distribution of communication material shaped the second and third phases of this project, enabling (for instance) a citizen science method to understand local GW characteristics via the technical outputs.

### 4.2. Groundwater flow and modeling

Our goal was to develop a citizen science method for monitoring GW, which we successfully implemented at the LH and HR sites (figures 2 and 3). GW wells were installed and monitored from April 19 to October 31, 2021, at LH and from May 14 to October 31, 2021, at HR, with the timeframe guided by the duration of our EPA funding. This period encompassed seasonal variations across spring, summer, and fall. While we measured GW at both LH and HR, we used LH as our primary example case for the hydrology measurements. Additionally, the mass balance approach is outlined in Teimoori *et al* (2021). To validate our method, we utilized a GW simulation model at LH, which provided a comprehensive understanding of the GW flow regime surrounding the green infrastructure installation.

**Figure 6.**
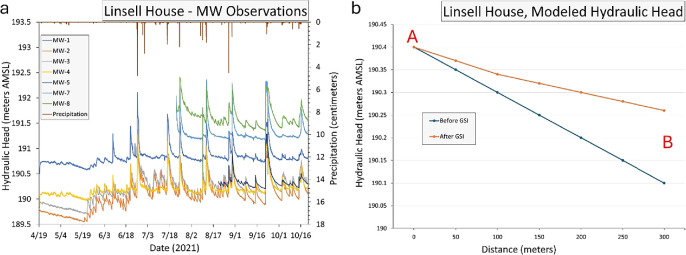
(A) Measured hydraulic head at Linsell House from April—October 2021 on left axis and daily precipitation on right axis and (B) modeled groundwater level changes before and after the installation of green stormwater infrastructure along line AB in figures 7(b) and (c).

**Figure 7.**
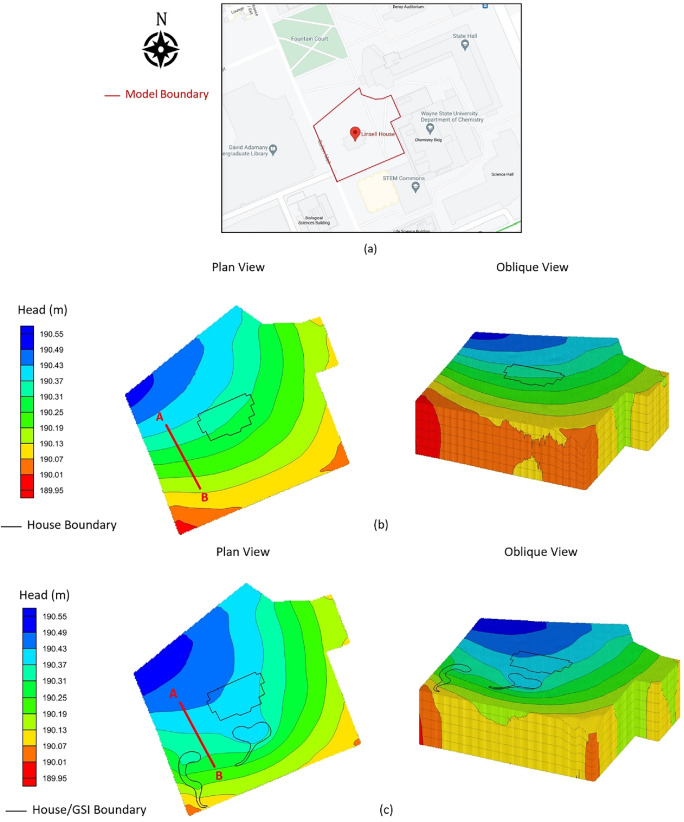
Simulation of groundwater regime at LH for the following conditions: (a) site map, (b) before GSI installation, and (c) after GSI installation. Black lines indicate LH footprint and GSI boundaries while the red line provides the cross-section used to present groundwater head changes in figure 6(b).

At LH, muted responses to rainfall events were observed in monitoring wells LH-1 through LH-4, while significant hydraulic head surges occurred within the GSI construction area (figure 6(a)). Similar hydraulic head surges were consistently observed at HR following substantial rainfall events (HR 1–4) (Supplementary figure 1). Interestingly, Phase 2 wells at LH (LH-5 through LH-8) exhibited response patterns comparable to those of wells adjacent to the GSI. It is notable that rainfall events occurred prior to construction, yet short-term variations in hydraulic head persisted beyond the summer and extended into the fall observation period. These hydraulic head fluctuations were also reflected in our GW model, which showed a consistent increase in hydraulic head following GSI installation (figure 7). Figure 6(b) provides a comparison of GW level elevations before and after GW installation. This paired-point analysis allows for a direct comparison of changes in GW head at identical points before and after the installation of the GSI. The data reflects how GW levels increased slightly after the installation, especially near the GSI, showing the infrastructure’s contribution to GW recharge (figure 6(b)). The *X*-axis represents the distance along a hypothetical transect line AB in figure 7. The modeled area encompasses a larger region, but this 300-meter section captures the key GW dynamics near and downstream of the GSI.

The model boundaries were defined as constant-head boundaries, with assigned parameters for conductance (0.025 m^2^/day/m) and hydraulic head (head stage) (189.8 to 190.6 meters) at model boundaries. Other important data assignments include recharge and evapotranspiration (ET) rates, set at 0.000524 m day^−1^ and 4.0e-006 m day^−1^, respectively. Supplementary table 1 summarizes key parameters assigned to the MODFLOW GW simulation of this site and Supplementary figure 2 graphically shows the 3D model layers.

The calibration process is critical to developing an accurate modeling tool for GW simulation. During the calibration process, the model is used to estimate a known solution (one that has been observed through measurements), and if the model output does not initially match the measured features, assigned parameters are systematically modified within a given range until the match between prediction and observation is within an acceptable value. In the present application, the most important output feature compared during the calibration process was GW table elevation (hydraulic head). Following calibration, the model could confidently simulate GW response (including change in GW table) to GSI installation. As shown in figure 7, the model simulations predicted that GW predominantly flowed from northwest to southeast before GSI installation. However, once the model included the GSI features, the simulation indicated a change in the distribution of the hydraulic head, which resulted in a local flow pattern. The GSI features were successful in altering the flow direction slightly toward the GSI, illustrating that the infrastructure was capturing stormwater and enhancing GW recharge. In turn, this increased the GW head levels around the site, reflecting the positive impact of the GSI on GW storage.

### 4.3. Groundwater and soil sampling

The LusterLeaf 1665 Professional Soil Kit for soil pH, nitrogen, phosphorus, and potassium was used for the first few sampling events; however, the test did not perform well due to the high clay content of the soil. Therefore, data derived from Hatch test kits (orthophosphate, PhosVer3; nitrate, NitraVer5; nitrite, NitriVer3) conducted on saturated soil extracts were used to determine nutrient concentrations in GW and soil.

Nitrite levels in soil samples collected from post-construction wells (MW 5–8), away from GSI at LH were significantly lower (*χ*^2^(2) = 6.86, p = 0.03) than those recorded in pre-construction (LH, MW 1–4) (p < 0.01) and reference wells (HR, MW 1–4) (p = 0.01). These wells had average nitrite concentrations of 0.001 ppm (SD ± 0.003), an order of magnitude lower than GSI-adjacent, pre-construction (M = 0.012 ppm, SD = 0.004), and reference wells (M = 0.010 ppm, SD ± 0.004). GW nitrite did not differ among well groups (*χ*^2^(2) = 5.21, p = 0.07).

Soil pH varied alongside nitrite (F(2,12) = 333.9, p < 0.01), with away wells having a lower pH (M = 5.13, SD ± 0.2) than pre-construction, GSI wells at LH (M = 6.13, SD ± 0.2) (p < 0.01) and reference wells at HR (M = 7.97, SD ± 0.07) (p < 0.01). Likewise, GW pH differed significantly between groups of wells (*χ*^2^(2) = 7.38, p = 0.02). However, unlike soil, GW pH levels were lowest in reference wells (M = 4.7, SD = 0.40) (p < 0.01) and did not differ by location when pre- (M = 5.8, SD ± 0.26) and post-GSI construction wells (M = 5.7, SD ± 0.25) were compared (p > 0.05).

GW temperature was significantly higher in well groups away from GSI, compared to pre-construction and reference wells (F(2,12) = 26.07, p < 0.01). GW collected from post-construction, away wells had an average temperature of 19.6 °C (SD ± 0.99), while pre-construction and reference wells had the same temperature, 16.5 °C (SD ± 0.38, ± 0.59, respectively).

Like the LusterLeaf soil testing products, our most affordable coliform testing strategy, 3M Petrifilm Microbial Indicator Plates, provided inconsistent results. Thus, Hatch m-ColiBlue24 broth was used to enumerate *E. coli* and total coliforms, while Difco™ mEI Agar was used to identify enterococci.

*E. coli* counts in soil samples were significantly lower in pre-construction, GSI adjacent wells at LH when compared to post-construction and reference wells (*χ*^2^(2) = 7.73, p = 0.02). At the reference site, and in post-construction wells away from GSI at LH, there was an average of 1935 (SD ± 773), and 1591 (SD ± 598) *E. coli* cells per sample, respectively. With an average of 269 (SD ± 199) *E. coli* cells per sample in GSI adjacent, pre-construction wells, these results indicate an increase of 591% (p < 0.01) in samples collected near post-GSI construction at LH and a 719% (p < 0.01) increase near reference wells at HR. This trend was not reflected in GW *E. coli* counts which did not differ significantly among groups of wells (*χ*^2^(2) = 1.88, p = 0.38).

Although soil total coliform counts did not differ between well groups, they did increase in pre-construction wells (LH, MW 1–4) over time (F_1,2_ = 63.4, R^2^ = 0.95, p = 0.01). This linear model indicated an average increase of 3972 total coliforms were detected per sample per month. Similarly, post-construction (LH, MW 5–8) data showed evidence of increasing coliform counts over time, but these results were not significant (F_1,1_ = 70.2, R^2^ = 0.97, p = 0.07). Total coliforms in GW did not change throughout the assessment, but *E. coli* (F_1,2_ = 13.2, R^2^ = 0.80, p = 0.06) and enterococci (F_1,2_ = 14.6, R^2^ = 0.82, p = 0.06) demonstrated a marginal trend towards statistical significance as cell counts increased over time.

There were no differences in nitrate and phosphorus levels, total coliform, enterococci MPs, conductivity, or dissolved oxygen concentrations in GW or soil when comparing pre/post-construction and reference wells (p > 0.05). Nor were there significant changes in nutrient analytes, MPs, or physicochemical parameters over time in pre-construction wells at LH.

## 5. Discussion

Overall, the community engagement, GW modeling, and GW sampling components revealed the ways in which a multidisciplinary research team can initiate partnerships within and outside of the university while developing a low-cost approach to observing and understanding GW in a neighborhood setting. The multiple pieces of this project provided an integrated platform to discuss an issue facing Detroit communities, which helped to deepen knowledge about urban water issues in both public and academic circles. Within this context, our approach to action research, the integrating research and action, was guided by our research and data collection and tailored to specific local conditions. Figure 1 presents a useful roadmap for others engaging in action research, with the inner circle outlining our main interactive and iterative framework and the surrounding boxes highlighting key variables and components used in our specific study. Our approach provides a structured framework and generates pragmatic insights for investigating relevant hydrological challenges within urban centers, as demonstrated in our example of Detroit.

Expanding knowledge about Detroit’s GW, in turn, provides critical data that improves the effectiveness of GSI installations and informs the development of an urban water budget, an important tool to assess water use availability and mitigation options for a given area. Simultaneously, furthering understanding about the role GSI can play in flooding issues has become relevant for the city. In the summer of 2021, when the bulk of our research was conducted, Detroit experienced its second 500-year flood event in seven years (Brooker 2021). While the modeling and sampling components of the project did not address flooding directly, the use of GSI is increasingly recognized as an effective method for managing stormwater, particularly in urban environments where impervious surfaces reduce natural infiltration (Kwak *et al* 2024). The results of the LH model demonstrate the potential for GSI to contribute to sustainable water management by not only mitigating surface runoff but also promoting GW recharge. By recharging GW, these systems help restore natural hydrological processes that are often disrupted in urban areas. The LH study offers insights into how bioswales and similar infrastructure can be integrated into urban planning efforts to address both water quality and quantity concerns. Moreover, the modeling approach used in this study can serve as a template for similar evaluations in other locations, particularly those with similar soil compositions or hydrological challenges.

This paper provided two approaches to understanding local urban water budgeting—a simple mass balance, and a complex three-dimensional modeling approach. The water budget equation offers a simple, straightforward approach to understanding the balance between water inputs and outputs in a system. This method is beneficial for simpler systems or scenarios where extensive spatial and temporal data are unavailable. It provides a clear, conceptual understanding of how various factors—such as precipitation, surface runoff, GW flow, and evapotranspiration—interact within the system. Cities such as Philadelphia, with its Green City, Clean Waters program (Baldridge *et al* 2016), and Los Angeles, through the Avalon Green Alleys Network (Sadeghi *et al* 2019), have employed similar methods, utilizing water level loggers and rain gauges to estimate GSI mass balances. While this approach is well-suited for initial assessments and cases where system complexity does not warrant advanced methods, it may lack the precision needed for more intricate or heterogeneous environments that require detailed temporal and spatial predictions. Conversely, advanced modeling software, such as MODFLOW, enables the simulation of complex hydrological processes with greater accuracy and specificity (Zhang and Chui 2020, Teimoori *et al* 2021). These models can integrate numerous variables, including subsurface heterogeneity, varying boundary conditions, and time-dependent changes, making them indispensable for dynamic systems such as groundwater-surface water interactions, the impacts of climate change, or contaminant transport in fractured media. Although these numerical models demand significant data input and computational resources, they provide a more detailed, predictive understanding of water movement and availability over space and time.

While changes to GW flow were noted, the changes to water quality revealed how landscape changes from GSI installations and introducing new plants to the system may have shifted the nutrient balance, resulting in increased soil nitrite levels near the new bioswale and rain garden installations at LH (Supplementary table 2). The transformation of nitrogenous compounds in soil is primarily controlled by two microbially mediated processes: nitrification and denitrification (Robertson and Groffman 2024). It is possible that long-established soil microbial communities were impacted by physicochemical changes imposed by plants (Meister *et al* 2023), causing nitrite, a product of incomplete nitrogen transformation, to build up in nearby soils (Supplementary table 1).

*E. coli* counts were lower in soils near new GSI installations at LH. Although *E. coli* is not a nitrogen-fixing bacterial species, it is sensitive to nitric oxide and nitrite in soil (Meng *et al* 2018). Soil chemistry and physicochemical parameters, which are known to affect microbial community composition and function (Meister *et al* 2023), may have impeded the proliferation of *E. coli* near GSI installations.

It has been long understood that soil nitrite transformations are inversely related to pH and temperature. Specifically, nitrogenous compounds denitrify at higher rates in acidic conditions (pH < 5.5) (Nelson and Bremner 1969) and warmer temperatures. This can volatilize nitric oxide and nitrite into gaseous compounds and destabilize soil nitrogen (Van Cleemput and Samater 1995). Cooler GW temperatures in wells proximal to GSI installations could lower soil temperatures, indirectly affecting soil pH and subsequent nitrite concentrations.

Total coliform counts in soil increased significantly near wells adjacent to new GSI installations at LH over time. These increases were dramatic, with our model suggesting nearly 4000 new cells in soil samples monthly. Similarly, *E. coli* and enterococci showed evidence of marginal increases in GW collected from wells each month. Further, soil nitrite data suggest a marginal decrease in concentrations over time. Although increasing seasonal temperatures could lead to higher levels of microbial metabolic activity and subsequent reproduction, it is possible that lower nitrite (and probable nitric oxide) concentrations opened an opportunity for microbial proliferation in adjacent soils to new GSI installations at LH.

### 5.1. Limitations and future perspectives

Our goal for this project was to source and implement affordable and accessible products that citizen science and community groups could employ to establish local GW monitoring programs (Supplementary table 3). The products we utilized included commercially available tests manufactured by LusterLeaf, Hach, Difco, and 3M. Although most of these products provided functional and reliable testing, nutrient tests from LusterLeaf (1665 Professional Soil Kit, Luster Leaf Rapitest Tester Electronic 4-Way Analyzer) were not useful for soil testing at our sites. The LusterLeaf products were the most affordable of the soil testing kits, but the high clay content in Detroit’s soil profile may have compromised the functionality of these tests (Faber *et al* 2007). Locations with different soil profiles may find these products suitable for their needs. We were able to test urban nutrient levels using soil extracts and Hach tests for soil nutrients (Hach Company 1982, Ormaza-González and Villalba-Flor 1994). These relatively inexpensive, over-the-counter nitrite, nitrate, and phosphate testing kits were helpful in analyzing nutrient levels in GW.

This study continued to explore affordable options to monitor soil and GW by using 3M Petrifilm Microbial Indicator Plates to quantify total coliforms, *E. coli*, and enterococci. 3M Petrifilm products are an effective method to assess microbial growth on food and environmental surfaces (Bird *et al* 2020). However, this product did not provide consistent results when used to test GW and soil extract. Like the nutrient analysis conducted in this study, Hatch products (m-ColiBlue24 broth) produced consistent and reliable results, as did Difco™ mEI Agar which was used to test enterococci. Although slightly more expensive than 3M Petrifilm, these products are designed to test environmental water samples making them more compatible with this study design. See Supplementary table 2 for a full list of products and prices utilized in this project.

Although there were statistically significant differences in nitrite concentrations and increases in total coliform counts in soils, and several GW and soil physicochemical parameters, many tested values were nearly significant (p < 0.1 > 0.05). Specifically, *E. coli*, enterococci, and NTU (turbidity) trended toward a significant linear regression model indicating possible increases over time. With a small sample size, nearly- and significant results, we can make two inferences. Firstly, the magnitude of the effect (testing of pre- and post-construction wells, their proximity to new GSI installations, and impacts over time) would have to be very large to be detected. Secondly, with the results of several parameters nearing significance, increasing the sample size may lower p-values, making differences in groups and linear relationships easier to identify (Thiese *et al* 2016).

The goal for the community engagement component was to develop communication material that local groups (CPC and ECN) could adopt in their education and outreach efforts with residents and policymakers. The resulting products, community discussions, and adoption, and the integration of community concerns into the design of the groundwater monitoring network and implementation of technical research were critical to the project’s impact and success. By providing an integrated STEAM (Science, Technology, Engineering, Arts, and Mathematics) approach to addressing urban GW concerns and producing methodologies that local citizen science groups can easily adopt, our project showcased effective and accessible solutions at the sociotechnical interface in line with the call articulated by the editors of this special issue. We also recognize the need to further validate our findings regarding community concerns. Therefore, future research may include focus groups or in-depth interviews with community members to further probe their concerns with urban flooding and the replacement of grey water infrastructure with green stormwater infrastructure. At the project’s outset, we planned to conduct detailed interviews with community members through online video calling, but the onset of the COVID-19 pandemic affected our ability to engage in public gatherings where we hoped to recruit participants for these remote interviews. Finally, although we reported back our educational products with our community partners, future research may consider evaluating the impact of such materials on community perceptions and concerns.

While frameworks for understanding and monitoring GSI exist, many continue to rely heavily on oversight or coordination from public sector agencies or academic institutions (Horner *et al* 2002, Spatari, *et al* 2011, Meixner *et al* 2021, Illinois Institute of Technology 2025). Although not exhaustive, the examples listed in Supplementary table 4 offer insight into current community-based GSI monitoring efforts and illustrate the range of engagement models in practice. Our framework is designed to enable a more resident-led approach—offering adaptable tools and strategies that equip community groups to initiate and sustain monitoring activities independently. By centering capacity-building and locally led science, our framework promotes a vision of community-oriented environmental monitoring that fosters autonomy and responsiveness to place-based priorities and concerns.

## 6. Conclusion

This project developed a network of community-based GW monitoring stations at GSI installations in two neighborhood sites in Detroit, MI. We also developed partnerships with community groups to increase knowledge about Detroit’s GW and GSI’s impact on GW resources. Our preliminary findings, including how GW levels increased after GSI installation and that nitrite levels and GW temperatures were lower at MWs closer to GSI, indicate the positive effect GSI has on GW quality and quantity. The data also provides a platform for further research on the city’s GW resources and provides an accessible tool to engage community residents in the process. By successfully implementing our design, we demonstrated how to establish a compact, low-cost network of GW monitoring stations to assess the impact of GSI on neighborhood-scale GW quantity and quality. The design of evidence-backed community engagement tools facilitated connections with nonprofit groups and residents impacted by flooding. The GW stations also allowed us to observe and measure microplastics, wastewater toxins, and employ low-cost technologies for rapid water quality detection. In this way, we highlight the importance of contributing to scientific knowledge about GW resources impacted by anthropogenic change, while sharing knowledge with local communities about the benefits of GSI in neighborhoods that need flooding mitigation technologies. Beyond flooding, these technologies may also be a vital tool for tracking contamination concerns across the city and identifying places where GSI installation may be relevant as a means of pollution mitigation from underground contaminants. Finally, this project exemplifies a productive collaboration between interdisciplinary undergraduate and graduate students from anthropology, biology, civil and environmental engineering, communication studies, and urban planning departments—as well as community nonprofit leaders, spanning social, economic, and environmental benefits.

## Data Availability

The data that support the findings of this study are openly available at the following URL/DOI: https://cfpub.epa.gov/ncer_abstracts/index.cfm/fuseaction/display.abstractDetail/abstract_id/11118/report/0.
